# Differentiation of Brain Tumor Recurrence from Post-Radiotherapy Necrosis with ^11^C-Methionine PET: Visual Assessment versus Quantitative Assessment

**DOI:** 10.1371/journal.pone.0132515

**Published:** 2015-07-13

**Authors:** Ryogo Minamimoto, Toshiyuki Saginoya, Chisato Kondo, Noriaki Tomura, Kimiteru Ito, Yuka Matsuo, Shigeo Matsunaga, Takashi Shuto, Atsuya Akabane, Yoko Miyata, Shuji Sakai, Kazuo Kubota

**Affiliations:** 1 Division of Nuclear Medicine, Department of Radiology, National Center for Global Health and Medicine, Tokyo, Japan; 2 Departments of Radiology, Southern Tohoku Research Institute for Neuroscience, Southern Tohoku General Hospital, Fukushima, Japan; 3 Department of Diagnostic Imaging and Nuclear Medicine, Tokyo Women's Medical University, Tokyo, Japan; 4 Research Team for Neuroimaging, Tokyo Metropolitan Institute of Gerontology, Tokyo, Japan; 5 Department of Neurosurgery, Yokohama Rosai Hospital, Yokohama, Kanagawa, Japan; 6 Department of Neurosurgery, NTT Medical Center Tokyo, Tokyo, Japan; 7 Department of Radiology, National Center for Global Health and Medicine Kohnodai Hospital, Chiba, Japan; University Hospital of Navarra, SPAIN

## Abstract

**Purpose:**

The aim of this multi-center study was to assess the diagnostic capability of visual assessment in L-methyl-^11^C-methionine positron emission tomography (MET-PET) for differentiating a recurrent brain tumor from radiation-induced necrosis after radiotherapy, and to compare it to the accuracy of quantitative analysis.

**Methods:**

A total of 73 brain lesions (glioma: 31, brain metastasis: 42) in 70 patients who underwent MET-PET were included in this study. Visual analysis was performed by comparison of MET uptake in the brain lesion with MET uptake in one of four regions (around the lesion, contralateral frontal lobe, contralateral area, and contralateral cerebellar cortex). The concordance rate and logistic regression analysis were used to evaluate the diagnostic ability of visual assessment. Receiver-operating characteristic curve analysis was used to compare visual assessment with quantitative assessment based on the lesion-to-normal (L/N) ratio of MET uptake.

**Results:**

Interobserver and intraobserver κ-values were highest at 0.657 and 0.714, respectively, when assessing MET uptake in the lesion compared to that in the contralateral cerebellar cortex. Logistic regression analysis showed that assessing MET uptake in the contralateral cerebellar cortex with brain metastasis was significantly related to the final result. The highest area under the receiver-operating characteristic curve (AUC) with visual assessment for brain metastasis was 0.85, showing no statistically significant difference with L/Nmax of the contralateral brain (AUC = 0.89) or with L/Nmean of the contralateral cerebellar cortex (AUC = 0.89), which were the areas that were the highest in the quantitative assessment. For evaluation of gliomas, no specific candidate was confirmed among the four areas used in visual assessment, and no significant difference was seen between visual assessment and quantitative assessment.

**Conclusion:**

The visual assessment showed no significant difference from quantitative assessment of MET-PET with a relevant cut-off value for the differentiation of recurrent brain tumors from radiation-induced necrosis.

## Introduction

Brain tumors overexpress a variety of l-amino acid transporters [1], and thus, l-methyl-^11^C-methionine (MET) is useful positron emission tomography (PET) tracer for imaging in neurooncology [2, 3]. The gadolinium (Gd)-enhanced magnetic resonance imaging (MRI) findings showing that radiation-induced necrosis mimics recurrent brain tumors are correct because of the breakdown of the blood-brain barrier in both states [4]. MET uptake in gliomas is closely related to both cellular proliferation [5] and the micro-vessel count [6]. The uptake of MET with recurrence due to tumor cells is different from that in radiation-induced injury where only passive diffusion across the broken blood-brain barrier occurs [7–10]. Therefore, MET-PET has the potential to differentiate recurrent brain tumors from post-radiotherapy necrosis.

The main quantitative value for differentiating between tumors and nontumoral lesions in MET-PET studies is the lesion-to-normal (L/N) background ratio. Herholz et al. showed that the optimal cut-off value was 1.47, providing a sensitivity of 76% and a specificity of 87% [2]. Terakawa et al. showed that the optimal cut-off value was different for metastatic brain tumors (L/Nmean: 1.41, sensitivity 79% and specificity 75%) and gliomas (L/Nmean: 1.58, sensitivity 75% and specificity 75%) [7]. Although MET-PET is useful for identifying tumor recurrence after radiation, the adopted L/N cut-off ratios as well as the type of standardized uptake value (SUV) used in calculating the ratio (mean or maximum) varies according to the nature of the primary lesion [7, 11].

O-(2-18F-fluoroethyl)-L-tyrosine (FET) with a cut off mean tumor-to-brain ratio of 1.95 showed a similar sensitivity (74%) and improved sensitivity (90%) for differentiation of recurrence of brain metastasis from radiation necrosis, compared to previous result on MET PET [12]. 3,4-Dihydroxy-6-[^18^F]fluoro-phenylalanine (^18^F-FDOPA) showed best sensitivity and specificity for diagnosis of recurrent GBM with T/N ratio of 1.3 [13].

Visual assessment has been a diagnostic method in PET studies. As Glaudemans et al. has suggested, visual assessment of MET-PET images may be easier to perform using criteria in which every area of uptake higher than background (normal gray matter) is considered potentially pathological [14].

The aim of our study was to assess the accuracy of visual assessment across centers, and to compare that accuracy to that of quantitative analysis.

## Materials and Methods

### Patients

The protocol for this retrospective observational study was approved by the institutional review board (IRB) of National Center for Global Health and Medicine and Tokyo Women's Medical University. The other participating facilities entrusted the institutional review board of National Center for Global Health with decisions on approval of the protocol for this retrospective observational study. All the IRB granted a waiver of consent for this retrospective observational study. Patient records and information were anonymized and de-identified prior to analysis.

The assessment was performed for seventy patients (38 males and 32 females; age range 26–85 years, mean 53.5 ± 14.3 years) with 73 lesions showing apparent contrast enhancement on follow-up gadolinium enhanced T1WI obtained by 1.5T MRI after intracranial irradiation (conventional radiotherapy and/or stereotactic radiosurgery). They were then imaged using MET-PET in one of three PET centers to discriminate a recurrent brain tumor from radiation-induced necrosis. The intracranial lesions were 42 metastatic brain tumors (primary lesions; lung: 27, breast: 9, colon: 1, sarcoma: 1, thyroid: 1, larynx: 1, squamous cell carcinoma: 1, kidney: 1) and 31 gliomas (anaplastic astrocytoma [grade 3]: 12, glioblastoma [grade 4]: 19). The locations of the lesions were 35 in the frontal lobe, 12 in the parietal lobe, 12 in the temporal lobe, 10 in the cerebellum, and 4 in the occipital lobe. All glioma cases were identified after surgery. Chemotherapy was employed in 27 cases of gliomas and was continuing for nine patients at the time of the MET-PET imaging. All patients had undergone conventional radiotherapy (n = 30, 50–60 Gy) or stereotactic radiosurgery (n = 40, 18–22 Gy) for gliomas or metastatic brain tumors.

Recurrence was defined as any of the following: 1) pathologic confirmation after tumor resection or biopsy, 2) death as the result of progression of the brain tumor disease, 3) increase in the size of the Gd-enhanced area on the following MRI where the lesion was matched to the MET uptake area, and 4) recurrence of the metastatic brain tumor was strongly suspected by the nuclear physician and shrinkage of the lesion was confirmed after additional radiation therapy. The defining characteristics of radiation-induced necrosis were clinical in nature. Such a lesion either remained stable or shrank in size without additional treatment as determined by a follow-up MRI (6 months or more after the PET scan). The mean interval between irradiation and the PET scan was 20.1 months for gliomas and 11.2 months for metastatic brain tumors. Final results were 48 cases of recurrent brain tumors and 25 cases of radiation-induced necrosis.

### MET-PET Imaging

Patients fasted for at least 3 h prior to the PET study. In PET center (A), the PET/CT examination was performed with a Discovery LS (GE Medical Systems, Milwaukee, WI) 20 min after intravenous injection of 220.8–738.8 MBq (mean 418.7 MBq) ^11^C-MET, using the 3D acquisition mode (n = 13) for a 10-min static scan. For attenuation correction, a non-enhanced CT scan was acquired, and attenuation-corrected images were reconstructed using the ordered subset expectation maximization (OSEM) algorithm. In PET center (B), 42 PET/CT examinations were performed with a Biograph 16 (Siemens, Erlangen, Germany) 20 min after injection of 370 MBq ^11^C-MET, using the 3D acquisition mode for a 10-min static scan. For attenuation correction, a non-enhanced CT scan was acquired, and attenuation-corrected images were reconstructed using OSEM. In PET center (C), 17 PET examinations were performed using an ECAT ACCEL (Siemens) 20 min after injection of 400 MBq ^11^C-MET, using the 3D acquisition mode for a 10-min static scan. ^68^Ge/^68^Ga sources were used for the transmission scan. At all centers, patients were placed in the scanner so that slices parallel to the orbitomeatal line could be obtained.

### PET Image Interpretation

#### Visual Analysis

A total of 73 lesions in 70 patients who underwent MET-PET were evaluated with visual assessment by three experienced, board-certified, nuclear medicine physicians. All reconstructed PET images were reviewed independently by the physicians on a work station, EV Insite (PSP Corporation, Tokyo, Japan). The lesion for visual assessment of the MET-PET image was determined by reference to the contrast-enhanced MRI of the brain. A visual assessment score for the MET-PET image was obtained by comparing MET uptake within the lesion to that in one of four specified areas: 1) the region surrounding the lesion, 2) the contralateral frontal lobe, 3) the entire contralateral region, and 4) the contralateral cerebellar cortex. The score was classified into five grades as follows: 1) much higher uptake, 2) slightly higher uptake, 3) almost the same uptake, 4) slightly lower uptake, and 5) much lower uptake. Finally, the results of visual assessment were reclassified into just two groups by means of consensus of three observers. The first group was “higher uptake than the reference region (grades 1 and 2)”, and the second group was “same or lower uptake than the reference region (grades 3, 4, and 5)”. To assess intraobserver reproducibility, these analyses were repeated by the same three observers approximately 3 months after the end of the first assessment. For analysis of the diagnostic value of visual assessment, we used a “consensus interpretation”, which was determined for each case as the choice selected by the majority of the readers.

#### Quantitative Analysis

The quantitative analysis was performed independently from the visual assessment. The region of interest (ROI) for lesions was manually located over the area corresponding to the contrast-enhanced area on the MRI. As a normal control, circular ROIs with a diameter of 10 mm were located over areas surrounding the lesion, within the gray matter of the contralateral frontal lobe, within the contralateral area, and within the contralateral cerebellar cortex.

The maximum SUV (SUVmax) and the mean SUV (SUVmean) were measured for the various ROIs, and the various L/N ratios were calculated by dividing the SUVmax of the lesion by the SUVmax of the normal control region (L/Nmax), by dividing the SUVmean of the lesion by the SUVmean of the normal control region (L/Nmean) and by dividing the SUVmax of the lesion by the SUVmean of the normal control region (L/Nmax mean)

### Statistical Analysis

Levels of interobserver agreement and intraobserver reproducibility were quantified using kappa values (κ-values) by two reclassified groups [15]. Bootstrapping was used to calculate 95% confidence intervals. The values for intraobserver reproducibility are shown as the average of the three readers. The result of visual assessments, which were sensitivity, specificity, positive predictive value (PPV), negative predictive value (NPV), accuracy, and the area under the receiver-operating characteristic curve (AUC) value based on receiver-operating characteristic (ROC) curve analysis, were calculated from the results of consensus interpretation. The average SUVmax and SUVmean for MET uptake and the L/N ratio were expressed as the means ± standard deviation (SD), and the Mann-Whitney U test was used to evaluate the differences in these values. The Kruskal-Wallis one-way analysis of variance was used to determine the difference in MET uptake and the L/N ratio among the three PET centers.

Multivariate logistic regression analysis was performed with “Assessment of the MET uptake in the lesion compared to each region” and the final result. ROC curve analysis was used to determine the optimal index of MET-PET and the cut-off values for differential diagnosis of tumor recurrence and radiation-induced necrosis. Differences in the AUC value among the reference regions for the lesion and between visual assessment and the quantitative value were compared using the test for the equality of ROC areas [16]. Statistical analyses were performed using the statistical package Stata (version IC 11; Stata Corp., TX, USA). Values of p < 0.05 were considered significant

## Results

### Visual analysis

Interobserver agreement and intraobserver reproducibility among the three readers for each index are summarized in Table 1.

**Table 1:** Intra-and interobserver variability.

Reference for lesion	Interobserver agreement			Intraobserver reproducibility		
	Kappa value (95% CI)			Kappa value (95% CI)		
	All	Glioma	Metastasis	All	Glioma	Metastasis
Around	0.568	0.591	0.541	0.662	0.685	0.642
	(0.422–0.710)	(0.081–0.860)	(0.340–0.694)			
Contralateral	0.568	0.557	0.564	0.633	0.610	0.651
	(0.524–0.625)	(0.323–0.649)	(0.551–0.651)			
Frontal lobe	0.532	0.536	0.507	0.694	0.657	0.718
	(0.466–0.568)	(0.499–0.588)	(0.279–0.650)			
Cerebellum	0.657	0.609	0.689	0.714	0.697	0.726
	(0.549–0.695)	(0.470–0.689)	(0.616–0.745)			

The values on intraobserver reproducibility were shown as averages of those in three readers.

The interobserver agreement was best when MET uptake in the tumor was compared to that in the contralateral cerebellar cortex (kappa = 0.657). The average intraobserver reproducibility was highest when MET uptake was compared to that in the contralateral cerebellar cortex. The result of visual analysis is shown in Table 2.

**Table 2:** Results of visual analysis.

Subject	Reference for lesion	Sensitivity	Specificity	PPV	NPV	Accuracy	AUC
All lesion	Around	91.5	53.8	78.2	77.8	78.1	0.73
	Contralateral	89.4	50.0	76.4	72.2	75.3	0.70
	Frontal lobe	85.1	57.7	78.4	68.2	75.3	0.71
	Cerebellum	78.7	73.1	84.1	65.5	76.7	0.76
Glioma	Around	85.7	50.0	78.3	62.5	74.2	0.68
	Contralateral	81.0	50.0	77.3	55.6	71.0	0.65
	Frontal lobe	71.4	60.0	78.9	50.0	67.7	0.66
	Cerebellum	66.7	60.0	77.8	46.2	64.5	0.63
Brain metastasis	Around	96.2	56.3	78.2	90.0	81.0	0.76
	Contralateral	96.2	50.0	75.8	88.9	78.6	0.73
	Frontal lobe	96.2	56.3	78.2	90.0	81.0	0.76
	Cerebellum	88.5	81.3	88.5	81.3	85.7	0.85

PPV: positive predictive value, NPV: negative predictive value, AUC: area under the receiver-operating characteristic curve.

Visual analysis showed relatively higher AUC values for evaluating recurrence of brain metastasis than for gliomas. For visual analysis of gliomas, no statistically significant difference in the AUC values was found among the reference regions for the lesion (p = 0.32–0.79). The cerebellum showed the highest AUC value for the assessment of brain metastasis. However, no significant difference was confirmed compared to the other references for the lesion (p = 0.07–0.16).

As summarized in Table 3, logistic regression analysis showed that the contralateral cerebellum was the most influential reference region for MET uptake for the final result in cases of brain metastasis (p = 0.03).

**Table 3:** Logistic regression analysis of the statistical significance (p value) of the dependence of the outcome on the visual assessment.

Reference region employed	P value		
	All lesions	Glioma	Metastasis
Around	0.24	0.31	0.99
Contralateral	0.96	0.89	0.99
Frontal lobe	0.84	0.87	0.99
Cerebellum	0.06	0.83	0.03

**Fig 1:**
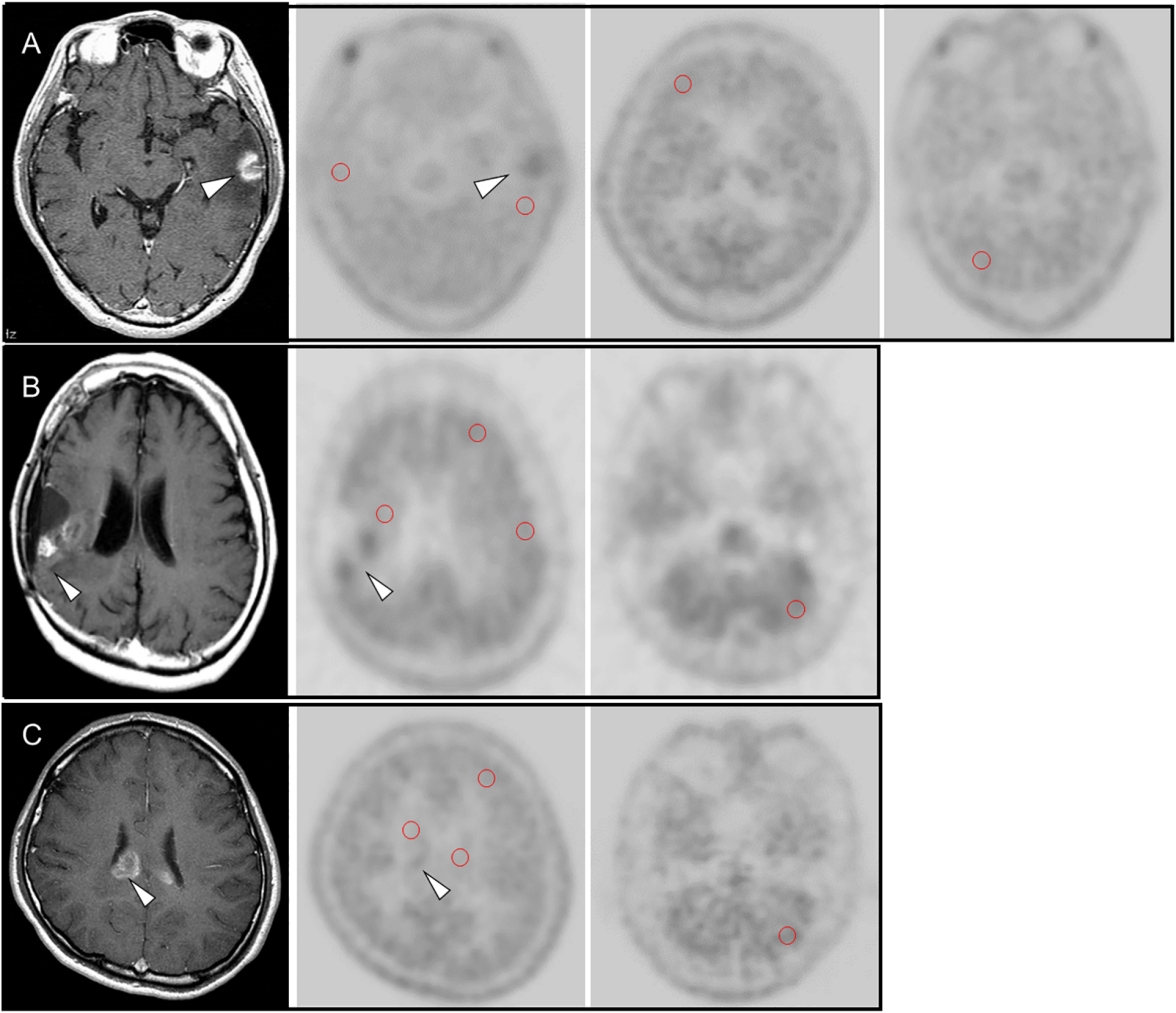
(A) 60-year-old male with recurrence of brain metastasis (lung carcinoma) at left temporal lobe (arrow head). MET uptake of the MRI-enhanced lesion showed higher than the region around the lesion, the contralateral brain, the contralateral frontal lobe and cerebellum. (B) 26-year-old male with recurrence of an anaplastic astrocytoma in the right temporal lobe. Contrast-enhanced MRI showed gadolinium-enhanced nodular lesion (arrow) in the right temporal lobe, which was the location of post-surgical resection of the primary brain tumor. MET uptake of the MRI-enhanced lesion (arrow head) showed higher than the region around the lesion, the contralateral brain, the contralateral frontal lobe and cerebellum. (C) 59-year-old female with radiation necrosis. Contrast-enhanced MRI showed gadolinium-enhanced lesion (arrow head) at the corpse callosum, which was suspected recurrent brain metastasis (lung carcinoma). MET uptake of the MRI-enhanced lesion showed slightly higher than the region around the lesion, the contralateral brain, and the contralateral frontal lobe, but similar to MET uptake at cerebellum.

However, no influential reference area was confirmed for the final result in cases of glioma. Representative MET-PET images are shown in Fig 1.

### Quantitative analysis

MET uptake for the cerebellum was significantly higher than for the other areas (p < 0.01), but no difference was found among the other areas (Table 4).

**Table 4:** Methionine uptake in normal brain areas and brain lesions.

Subject	SUVmax (± SD)	Range	SUVmean (±SD)	Range
Around	1.4 ± 0.4	0.7–2.2	1.1 ± 0.3	0.4–1.8
Contralateral	1.5 ± 0.4	0.6–2.8	1.2 ± 0.3	0.6–2.7
Frontal lobe	1.4 ± 0.3	0.5–2.2	1.2 ± 0.2	0.5–1.8
Cerebellum	1.7 ± 0.4	0.7–2.5	1.5 ± 0.3	0.7–2.3
Recurrence	2.5 ± 0.9	0.7–6.3	2.0 ± 0.8	0.6–5.6
Glioma	2.5 ± 1.1	0.7–6.3	2.1 ± 1.0	0.6–5.6
Metastasis	2.5 ± 0.8	1.3–4.1	2.0 ± 0.6	0.7–3.3
Radiation necrosis	1.9 ± 0.9	0.7–4.7	1.5 ± 0.7	0.6–4.0

SUVmax: maximum standardized uptake value, SUVmean: mean standardized uptake value, SD: standardized deviation.

The SUV was significantly higher for tumor recurrence than for radiation-induced necrosis (SUVmax: p < 0.006, SUVmean: p < 0.005). The SUVmax and SUVmean of gliomas were not different from those of metastasis (p = 0.35). Each of the three L/N ratios using the cerebellum as a reference was significantly lower than when using the other region as a reference (p < 0.01) (Table 5).

**Table 5:** Lesion-to-normal tissue ratio.

Subject	Reference to lesion	L/N max	L/N mean	L/N max mean
Recurrence (Glioma and metastasis)	Around	1.7 ± 0.6 (1.0–3.9)	1.8 ± 0.8 (0.6–4.4)	2.2 ± 1.0 (1.0–5.0)
	Contralateral	1.8 ± 0.7 (0.6–5.6)	1.7 ± 0.8 (0.5–6.0)	2.1 ± 0.9 (0.6–6.8)
	Frontal lobe	1.9 ± 0.7 (0.9–5.1)	1.8 ± 0.8 (0.6–5.4)	2.1 ± 0.9 (1.1–6.1)
	Cerebellum	1.5 ± 0.5 (0.7–3.8)	1.4 ± 0.5 (0.5–3.6)	1.7 ± 0.6 (0.8–4.1)
Glioma	Around	1.6 ± 0.7 (1.0–3.9)	1.7 ± 0.8 (0.9–4.4)	2.0 ± 0.9 (1.0–5.0)
	Contralateral	1.8 ± 1.0 (0.6–5.6)	1.9 ± 1.1 (0.5–6.0)	2.1 ± 1.2 (0.6–6.8)
	Frontal lobe	1.8 ± 0.9 (0.9–5.1)	1.8 ± 1.0 (0.8–5.4)	2.0 ± 1.0 (1.1–6.1)
	Cerebellum	1.5 ± 0.7 (0.7–3.8)	1.4 ± 0.7 (0.7–3.6)	1.6 ± 0.7 (0.8–4.1)
Metastasis	Around	1.8 ± 0.5 (1.0–3.0)	1.9± 0.8 (0.6–4.2)	2.5 ± 1.0 (1.2–5.0)
	Contralateral	1.7 ± 0.4 (0.9–2.8)	1.6 ± 0.5 (0.6–3.2)	2.1 ± 0.6 (1.2–3.8)
	Frontal lobe	1.9 ± 0.6 (1.0–3.6)	1.8 ± 0.6 (0.6–3.3)	2.3 ± 0.7 (1.2–4.0)
	Cerebellum	1.5 ± 0.4 (0.8–2.5)	1.4 ± 0.4 (0.5–2.4)	1.7 ± 0.5 (0.9–2.8)
Radiation necrosis	Around	1.3 ± 0.4 (0.6–2.1)	1.3 ± 0.4 (0.6–2.0)	1.8 ± 0.9 (0.7–4.9)
	Contralateral	1.2 ± 0.3 (0.6–2.3)	1.2 ± 0.4 (0.7–2.4)	1.6 ± 0.6 (0.5–3.8)
	Frontal lobe	1.3 ± 0.4 (0.6–2.4)	1.2 ± 0.4 (0.6–2.3)	1.7 ± 0.9 (0.7–5.0)
	Cerebellum	1.0 ± 0.3 (0.5–1.8)	0.9 ± 0.3 (0.4–1.8)	1.3 ± 0.7 (0.5–3.8)

Range is represented in parenthesis. L/N ratio: lesion to normal tissue ratio L/Nmax: SUVmax (lesion) / SUVmax (reference), L/Nmean: SUVmean (lesion)/ SUV mean (reference), L/N max mean: SUVmax (lesion)/ SUV mean (reference).

The L/N ratio was significantly higher for tumor recurrence than for radiation-induced necrosis (p < 0.02).

### Variation in MET uptake among PET centers

The results for variation in MET uptake among PET centers are shown in Tables 6 and 7.

**Table 6:** Differences in SUV among the three PET centers.

Subject	SUVmax				SUVmean			
	A	B	C	P value	A	B	C	P value
Around	1.7 ± 0.3	1.3 ± 0.3	1.4 ± 0.3	0.02	1.3 ± 0.3	1.1 ± 0.3	1.2 ± 0.3	0.06
Contralateral	1.7 ± 0.3	1.4 ± 0.3	1.4 ± 0.5	0.06	1.3 ± 0.3	1.2 ± 0.3	1.3 ± 0.5	0.32
Frontal lobe	1.7 ± 0.3	1.3 ± 0.2	1.3 ± 0.3	<0.001	1.4 ± 0.3	1.1 ± 0.2	1.2 ± 0.3	<0.001
Cerebellum	2.1 ± 0.3	1.6 ± 0.3	1.6 ± 0.3	<0.001	1.8 ± 0.3	1.4 ± 0.3	1.5 ± 0.3	<0.001
Lesions	2.2 ± 0.5	2.4 ± 1.1	1.9 ± 0.7	0.28	1.6 ± 0.4	1.9 ± 1.0	1.7 ± 0.6	0.26

A: PET cancer (A), B: PET cancer (B), C: PET cancer (C).

**Table 7:** Differences in the L/N ratio in cases with radiation-induced necrosis among the three PET centers.

Subject	L/N max				L/N mean				L/N max mean			
	A	B	C	P value	A	B	C	P value	A	B	C	P value
Around	1.2 ± 0.2	1.3 ± 0.4	1.4 ± 0.3		1.2 ± 0.2	1.4 ± 0.4	1.4 ± 0.4		1.7 ± 0.4	1.8 ± 0.7	1.7 ± 0.5	
Contralateral	1.2 ± 0.3	1.3 ± 0.4	1.2 ± 0.2	0.36	1.2 ± 0.4	1.2 ± 0.4	1.1 ± 0.2	0.71	1.5 ± 0.2	1.6 ± 0.6	1.6 ± 0.4	0.46
Frontal lobe	1.2 ± 0.4	1.4 ± 0.4	1.3 ± 0.3	0.40	1.1 ± 0.4	1.2 ± 0.4	1.2 ± 0.3	0.55	1.6 ± 0.2	1.7 ± 0.9	1.6 ± 0.5	0.99
Cerebellum	1.0 ± 0.3	1.1 ± 0.3	1.1 ± 0.2	0.38	0.8 ± 0.3	1.0 ± 0.3	0.9 ± 0.3	0.45	1.2 ± 0.1	1.2 ± 0.6	1.3 ± 0.4	0.66

A: PET cancer (A), B: PET cancer (B), C: PET cancer (C), L/N ratio: lesion to normal tissue ratio L/Nmax: SUVmax (lesion) / SUVmax (reference), L/Nmean: SUVmean (lesion)/ SUV mean (reference), L/N max mean: SUVmax (lesion)/ SUV mean (reference).

**Fig 2:**
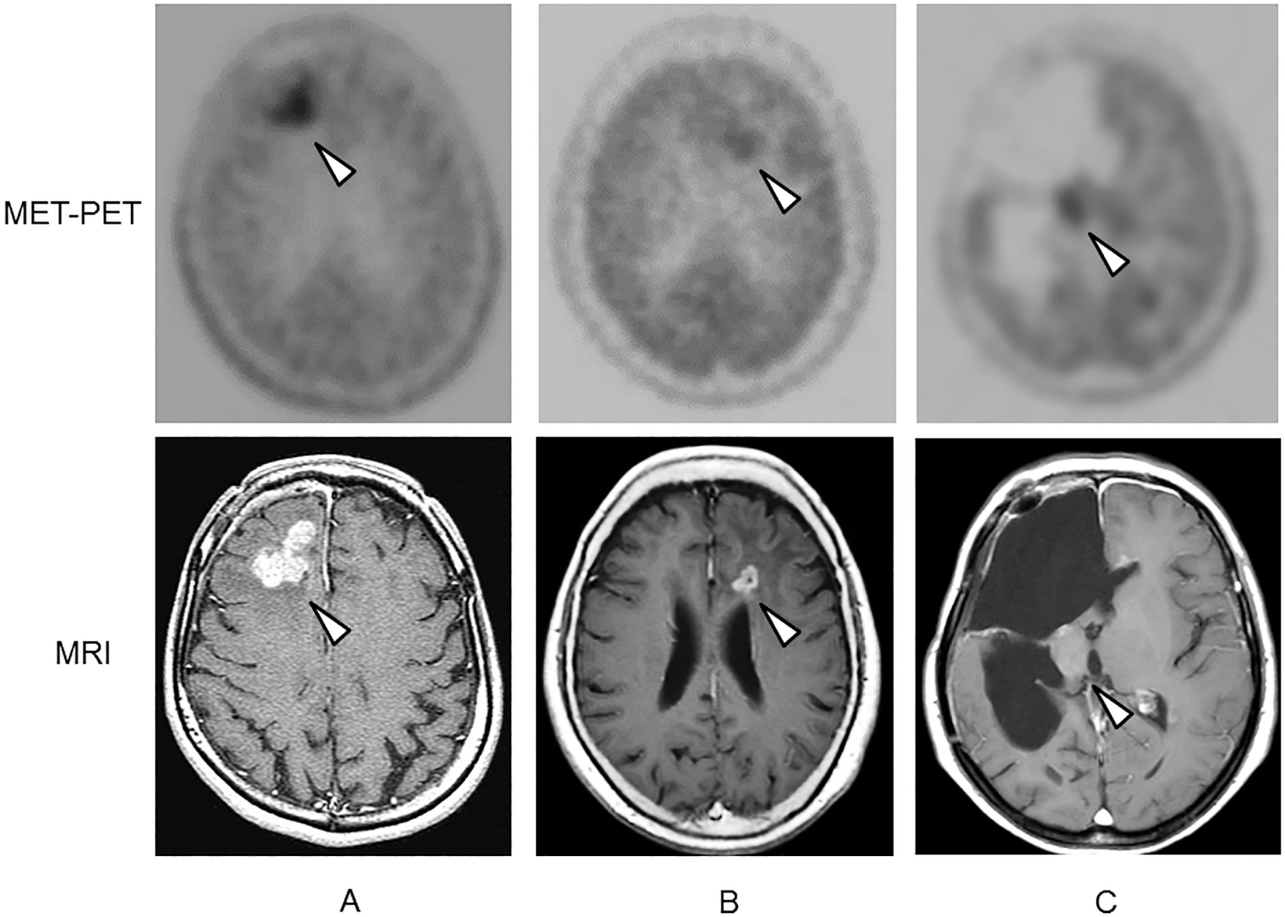
Representative methionine PET (MET-PET) images from the three PET centers (A, B, and C) and reference contrast-enhanced MRIs from patients with recurrent gliomas. All lesions are indicated with arrow heads.

Significant differences in MET uptake around the lesion, in the contralateral frontal lobe, and in the contralateral cerebellum were confirmed among the three PET centers. However, no significant difference was confirmed in the L/N ratio for cases with radiation-induced necrosis among the three PET centers. Representative MET-PET images are shown in Fig 2.

### ROC analysis for distinguishing tumor recurrence from radiation-induced necrosis

The assessment using a cut-off value for the L/N ratio is shown in Table 8.

**Table 8:** The cut-off value for the L/N ratio based on ROC analysis.

Subject		All lesions		Glioma		Metastasis	
	SUV	L/N ratio*	AUC	L/N ratio*	AUC	L/N ratio*	AUC
Around	Max	1.4	0.72	1.5	0.63	1.4	0.79
	Mean	1.3	0.70	1.3	0.61	1.3	0.76
	Max mean	1.7	0.68	1.8	0.69	1.6	0.71
Contralateral	Max	1.3	0.79	1.4	0.68	1.3	0.89
	Mean	1.3	0.77	1.4	0.66	1.3	0.85
	Max mean	1.7	0.68	1.8	0.59	1.6	0.66
Frontal lobe	Max	1.5	0.72	1.2	0.60	1.5	0.81
	Mean	1.4	0.78	1.2	0.60	1.4	0.88
	Max mean	1.6	0.74	1.4	0.63	1.6	0.84
Cerebellum	Max	1.2	0.79	1.0	0.60	1.2	0.86
	Mean	1.1	0.81	1.2	0.71	1.1	0.89
	Max mean	1.4	0.75	1.2	0.59	1.3	0.83

Results for the four reference regions, three types of L/N, and two types of patient as well as for all patients. L/N ratio: lesion to normal tissue ratio, SUV: standardized uptake value, Max: SUVmax (lesion) / SUVmax (reference), Mean: SUVmean (lesion)/ SUV mean (reference), L/N max mean: SUVmax (lesion)/ SUV mean (reference), AUC: area under the receiver-operating characteristic curve.

*The number shown in L/N ratio is cut off value based on ROC analysis.

The AUC value was highest for L/Nmean using the cerebellum as a reference (AUC = 0.81), but no significant difference was confirmed among the patients. For distinguishing gliomas from radiation-induced necrosis, the L/Nmean with the cerebellum as a reference showed the highest AUC value (AUC = 0.71). For distinguishing metastasis from radiation-induced necrosis, the L/Nmax with the contralateral area as a reference (AUC = 0.89) and the L/Nmean with the cerebellum as a reference (AUC = 0.89) showed the highest AUC values.

### Comparing visual and quantitative analysis

The AUC value with visual observation was 0.76, which had no significant difference with with the L/Nmean of the contralateral cerebellum (AUC = 0.81). For evaluating gliomas, no statistically significant difference was confirmed between visual assessment using the area around the lesion as a reference (AUC = 0.68) and quantitative analysis using the L/N cut-off value with the contralateral cerebellum as a reference. For evaluating metastasis, no statistically significant difference was confirmed between visual assessment using the contralateral cerebellum as a reference (AUC = 0.85) and quantitative analysis using L/N cut-off values of 1.3 with the contralateral side as a reference (AUC = 0.89) and 1.1 with the contralateral cerebellum as a reference (AUC = 0.89) (Table 8). The results of quantitative analysis with the two highest AUC values are shown in Table 9.

**Table 9:** Results of quantitative analysis.

Lesion	Subject	SUV	Cut off value	Sensitivity	Specificity	PPV	NPV	Accuracy	AUC
All lesions	Contralateral	Max	1.3	75.0	72.0	83.7	60.0	74.0	0.79
	Cerebellum	Mean	1.1	67.8	84.0	58.2	58.3	74.0	0.81
Glioma	Contralateral	Max	1.4	66.7	60.0	77.8	46.2	64.5	0.68
	Cerebellum	Mean	1.2	52.4	90.0	91.7	47.4	64.5	0.71
Metastasis	Contralateral	Max	1.3	81.5	85.7	91.7	72.0	83.3	0.89
	Cerebellum	Mean	1.1	81.5	86.7	91.7	72.0	83.3	0.89

AUC: area under the receiver-operating characteristic curve.

The quantitative value showed a lower sensitivity and higher specificity than visual analysis, although no significant difference in the AUC values was found. As a consequence, no significant difference between quantitative evaluation and visual analysis was identified.

## Discussion

The results from this multi-center study showed that visual assessment was not significantly different from quantitative assessment of MET-PET with a relevant cut-off value for the differentiation of recurrent brain tumors from radiation-induced necrosis.

The L/N ratio has been used for differentiation, but the cut-off ratio is different in each study [7, 8, 17–19]. The variation may be caused by several factors within the PET scan protocol, which has not been standardized. No studies have assessed observer variability in MET-PET for differentiation of recurrent brain tumors from radiation-induced necrosis. Therefore, we expected that visual assessment may solve the problem.

The cerebellum appeared to be the best candidate reference area for visual assessment because of high interobserver agreement, intraobserver reproducibility, and specificity. This region may be useful only for cases of suspected recurrence of brain metastasis. However, the low sensitivity of the cerebellum as a reference for lesions was a serious limitation for visual assessment. We did not find a relevant reference area for assessing gliomas, although the region around the lesion showed the best value for visual analysis. Unfortunately, a combination of these parameters for assessment did not improve the diagnosis (data not shown).

Distribution of normal MET uptake, which is obtained by recalculating the images as ratios to whole-brain mean uptake, is higher in the occipital cortex, cerebellum, and thalamus than in other regions [20]. In our study, quantitative analysis showed the same trend. A wide variation in MET uptake appeared to be confirmed in the occipital cortex, cerebellum, basal ganglia, and brain stem [20]. In contrast, uptake in the cortex, except the occipital area and white matter, showed low variation. MET uptake in the thalamus is similar to that in the cerebrum and shows little variation. Therefore, the thalamus is a candidate reference area, but the small area of this region made reliable identification difficult, and thus, we did not include it as a reference area in this study.

A remarkable point is that the results of visual assessment were different for gliomas compared to brain metastases. The cerebellum is the most influential region for differentiating between recurrence of brain metastasis and radiation-induced necrosis. The present study showed a wide range of MET uptake for glioma lesions (range of SUVmax: 0.7–6.3) and a relatively higher SD (1.1) than metastasis (0.8). Another assumed cause of the wide range with MET uptake for glioma lesion is that most of the cases we included were in the post-operative state. According to the quantitative result, MET uptake for glioma lesions was similar to that for metastatic lesions. Nevertheless, the L/N ratio was higher in metastatic lesions than gliomas, indicating that several unknown factors regarding gliomas and the variation in MET uptake for these lesions may have affected the result. In several articles evaluating MET-PET for differentiation of recurrence from radiation-induced necrosis, the mean L/N ratio was higher in gliomas than in brain metastases.

Our results indicate that the quantitative result was not significantly different from visual assessment. The problem with quantitative assessment is determining the cut-off value. Each facility will be able to estimate the cut-off value using retrospective observation analysis and matching of the L/N ratio for each MET-PET image and the patient’s prognosis or intervening pathological diagnosis. According to our result, we expected that visual assessment may be useful as a brief reference for estimating the range of cut-off values.

High-dose radiation therapies and repeated radiotherapies prolong patient survival, but they also increase the incidence of radiation-induced necrosis [21, 22]. In this study, MET uptake was assessed with reference to Gd-enhanced lesions seen on MRI. Symptomatic brain edema occurred in both recurrent tumors and radiation-induced necrosis. Therefore, assessment of brain edema using Gd-enhanced T1-weighted MRI as references should also be included when evaluating the patient after radiotherapy.

^18^F-FET PET can provide valuable information for differentiation of high-grade glioma or brain metastasis from treatment-related changes in the brain tissue [12, 23]. In addition to the T/B ratio of ^18^F-FET uptake, ^18^F-FET kinetics showed potential diagnostic value for the differentiation [12].

Recently, bevacizumab was reported to be effective for improving perilesional edema around the necrotic core, although this drug cannot induce functional recovery of necrotic tissue [24–26]. ^18^F-boronophenylalanine, which is an amino acid tracer similar to MET, is useful for diagnosing radiation-induced necrosis and predicting the efficacy of bevacizumab in progressive radiation-induced necrosis [27].

A limitation of this study is that it is a retrospective study, and we could not obtain histological confirmation of all cases at the time of MET-PET to differentiate recurrence from radiation-induced necrosis. All patients in this study underwent radiation therapy after pathological confirmation after a surgical procedure or clinical diagnosis based on film evidence. Therefore, clinical follow-up is valid in cases of suspected recurrence and has the ethical advantage of avoiding an invasive procedure such as a biopsy. MET-PET is an acceptable method for assessing CNS tumors as it avoids invasive procedures.

This study included only patient with grade III and IV glioma, it might cause a bias in this study. The lack of PET image quality control across the three facilities is another limitation of this retrospective study, although the L/N ratio was not significantly different among the three PET centers.

## Conclusion

The results of this multicenter study show that the cerebellum is the best candidate for visual assessment for distinguishing recurrence of brain metastasis from radiation-induced necrosis because of the high interobserver agreement, intraobserver reproducibility, and AUC value. MET uptake in a suspected lesion, compared to uptake in the contralateral cerebellum, was significantly related to the final result. Quantitative values were not significantly different from visual assessment for differentiating recurrent brain tumors from radiation-induced necrosis.
